# Risk factors, outcomes, and epidemiological and etiological study of hospitalized COVID-19 patients with bacterial co-infection and secondary infections

**DOI:** 10.1007/s10096-024-04755-5

**Published:** 2024-01-22

**Authors:** Yishan Duan, Jing Wang, Suyan Wang, Rui Zhang, Jinrui Hu, Weimin Li, Bojiang Chen

**Affiliations:** 1 Department of Respiratory and Critical Care Medicine, West China Hospital of Sichuan University, Chengdu, 610041 Sichuan Province China; 2 Precision Medicine Center, Precision Medicine Key Laboratory of Sichuan Province, West China Hospital, Sichuan University, Chengdu, 610041 China; 3 Frontiers Science Center for Disease-Related Molecular Network, West China Hospital, Sichuan University, Chengdu, 610041 Sichuan Province China; 4 The Research Units of West China, Chinese Academy of Medical Sciences, West China Hospital, Chengdu, 610041 Sichuan China; 5 State Key Laboratory of Respiratory Health and Multimorbidity, West China Hospital, Chengdu, 610041 Sichuan China

**Keywords:** COVID-19, Risk factors, Outcomes

## Abstract

**Background:**

As a common complication of viral respiratory tract infection, bacterial infection was associated with higher mortality and morbidity. Determining the prevalence, culprit pathogens, outcomes, and risk factors of co-infection and secondary infection occurring in hospitalized patients with coronavirus disease 2019 (COVID-19) will be beneficial for better antibiotic management.

**Methods:**

In this retrospective cohort research, we assessed clinical characteristics, laboratory parameters, microbiologic results, and outcomes of laboratory-confirmed COVID-19 patients with bacterial co-infection and secondary infection in West China Hospital from 2022 December 2nd to 2023 March 15th.

**Results:**

The incidence of bacterial co-infection and secondary infection, as defined by positive culture results of clinical specimens, was 16.3% (178/1091) and 10.1% (110/1091) respectively among 1091 patients. *Acinetobacter*, *Klebsiella*, and *Pseudomonas* were the most commonly identified bacteria in respiratory tract samples of COVID-19 patients. In-hospital mortality of COVID-19 patients with co-infection (17.4% vs 9.5%, *p* = 0.003) and secondary infection (28.2% vs 9.5%, *p* < 0.001) greatly exceeded that of COVID-19 patients without bacterial infection. Cardiovascular disease (1.847 (1.202–2.837), *p* = 0.005), severe COVID-19 (1.694 (1.033–2.778), *p* = 0.037), and critical COVID-19 (2.220 (1.196–4.121), *p* = 0.012) were proved to be risk factors for bacterial co-infection, while only critical COVID-19 (1.847 (1.202–2.837), *p* = 0.005) was closely related to secondary infection.

**Conclusions:**

Bacterial co-infection and secondary infection could aggravate the disease severity and worsen clinical outcomes of COVID-19 patients. Notably, only critical COVID-19 subtype was proved to be an independent risk factor for both co-infection and secondary infection. Therefore, standard empirical antibiotics was recommended for critically ill COVID-19 rather than all the inpatients according to our research.

**Supplementary Information:**

The online version contains supplementary material available at 10.1007/s10096-024-04755-5.

## Introduction

As a new acute respiratory infectious disease caused by severe acute respiratory syndrome coronavirus 2 (SARS-CoV-2), coronavirus disease 2019 (COVID-19) has resulted in more than 700 million confirmed cases and approximately 7 million deaths have been reported globally [1]. It is widely known that bacterial infection is the most common complication of viral respiratory tract infection. The pathogenesis of bacterial invasion in the setting of severe viral infection has not been defined yet, and disruption of epithelial barrier function, dysregulation of immune function, and inflammatory response may promote this process [2–4]. Patients infected with common respiratory viral pathogens, such as influenza, rhinovirus, and Middle East respiratory syndrome coronavirus (MERS-CoV), have been reported to be susceptible to bacterial invasion and the incidence of bacterial infection varies among different viral pathogens [2, 5, 6]. In addition, bacterial infection is widely considered to be associated with worse clinical outcomes in patients with viral respiratory tract infections [5, 6]. At present, the susceptibility of bacterial infection in COVID-19 patients remains unknown and reported bacterial infection rate varies among different study populations [7, 8].

The main clinical manifestations of COVID-19 patients included fever, cough, and sore throat, accompanied by lung imaging abnormalities [9]. Severe COVID-19 are characteristic by uncontrolled inflammatory responses and cytokine storm-like syndromes, so abnormally increased inflammatory biomarkers and cytokines (e.g., procalcitonin (PCT), C-reactive protein (CRP), interleukin-6 (IL-6), IL-1β, IL-1RA, IL-17A, and tumor necrosis factor-α (TNF-α)) also were observed in COVID-19 patients without bacterial infection [10–13]. Similar signs and symptoms bring immense difficulty to differential diagnosis between COVID-19 and bacterial pneumonia, so empiric antibiotics were prescribed in over three-quarters of hospitalized COVID-19 patients in reported studies. However, antibiotics provide no benefit for COVID-19 treatment, yet it significantly multiplies the global burden of antibiotic resistance [14, 15].

Therefore, we aimed to carry out a research concerning the incidence of bacterial co-infection and secondary infection among hospitalized COVID-19 patients and document the detailed culprit pathogens to define disease burden, which will be essential to optimize clinical antibiotic management and minimize antibiotic overuse. Besides, we also full analyzed the impact of co-infection and secondary infection on the clinical outcome of SARS-CoV-2 infection. Lastly, risk factors for bacterial co-infection and secondary infection were further explored to develop effective prevention strategies.

## Method

A retrospective cohort study was performed in West China Hospital of Sichuan University between December 2nd, 2022, and March 15th, 2023. We recruited all patients hospitalized with laboratory-confirmed COVID-19, which was identified by positive real-time reverse transcription polymerase chain reaction (RT-PCR) or rapid antigen test results from an oronasopharyngeal swab for SARS-CoV-2 infection before hospitalization admission. Patients were excluded if: (1) aged < 16 years old, (2) patients discharged within 48 h of admission; (3) patients who did not take bacterial culture during the hospital stay, including unqualified (in sputum specimen, squamous epithelial cells ≤ 10/low-power field (LPF) and white blood cells (WBC) ≥ 25/LPF are regarded as qualified sputum specimen of the lower respiratory tract) or contaminated clinical specimen; (4) asymptomatic COVID-19 who were admitted for other diseases (e.g., for surgery); (5) chest computed tomography (CT) showed no infiltration.

Bacterial infections were diagnosed by both typical clinical symptoms and positive bacterial culture of respiratory tract samples, blood, or other clinical specimens (including urine, pleural fluid, and cerebrospinal fluid). Bacterial infection among COVID-19 patients was divided into co-infection and secondary infection according to timing from the time from hospitalization to pathogen isolation. Criteria for bacterial co-infection were defined as a positive bacterial culture of clinical specimens identified within 48 h of admission, meaning in the presence of a positive COVID-19 PCR or antigen test. Secondary infections were identified by a bacterial infection that developed after admission of more than 48-h duration.

We retrieved demographic (age, gender), BMI, former or current smoker, comorbidities (including diabetes, hypertension, chronic kidney/hepatic/lung diseases, cardiovascular disease, immune system disease, and malignancy), laboratory parameters at admission, and microbiologic results (the proportion of patients with co-infections and secondary infections, the strains isolated from positive samples), antibiotics prescription records, and clinical outcome from electronic medical record. All patients were followed until death in hospital or hospital discharge. The clinical outcome includes the length of hospital stays, the need for tracheal intubation and mechanical ventilation (including tracheal intubation and non-invasive ventilator), ICU admission, and all-cause mortality during hospitalization. COVID-19 was classified as critical COVID-19, severe COVID-19, and non-severe COVID-19 according to WHO definitions of disease severity [9].

## Statistical analyses

Continuous variables were presented as means ± standard deviations (SD) and categorical variables were expressed as absolute numbers and frequencies (%) respectively. Mann–Whitney *U* test, chi-square test, Fisher exact test, and independent‐samples *T*-test were used to compare differences among patients without bacterial infection, patients with bacterial co-infections, or secondary infections depending on the data. The effect of covariates on bacterial co-infections and secondary infections among hospitalized COVID-19 patients were further assessed by odds ratios (OR) and 95% confidence intervals (95% CI) in a multivariable logistic regression (MLR) model. Variables with *p* ≤ 0.1 in a univariate analysis were included in the model.

For all comparisons, differences were tested using two-tailed tests, and statistical significance was set at *p* < 0.05. Statistical analyses were performed by SPSS Statistics Version 26.0 (Armonk, NY).

## Result

**Fig. 1:**
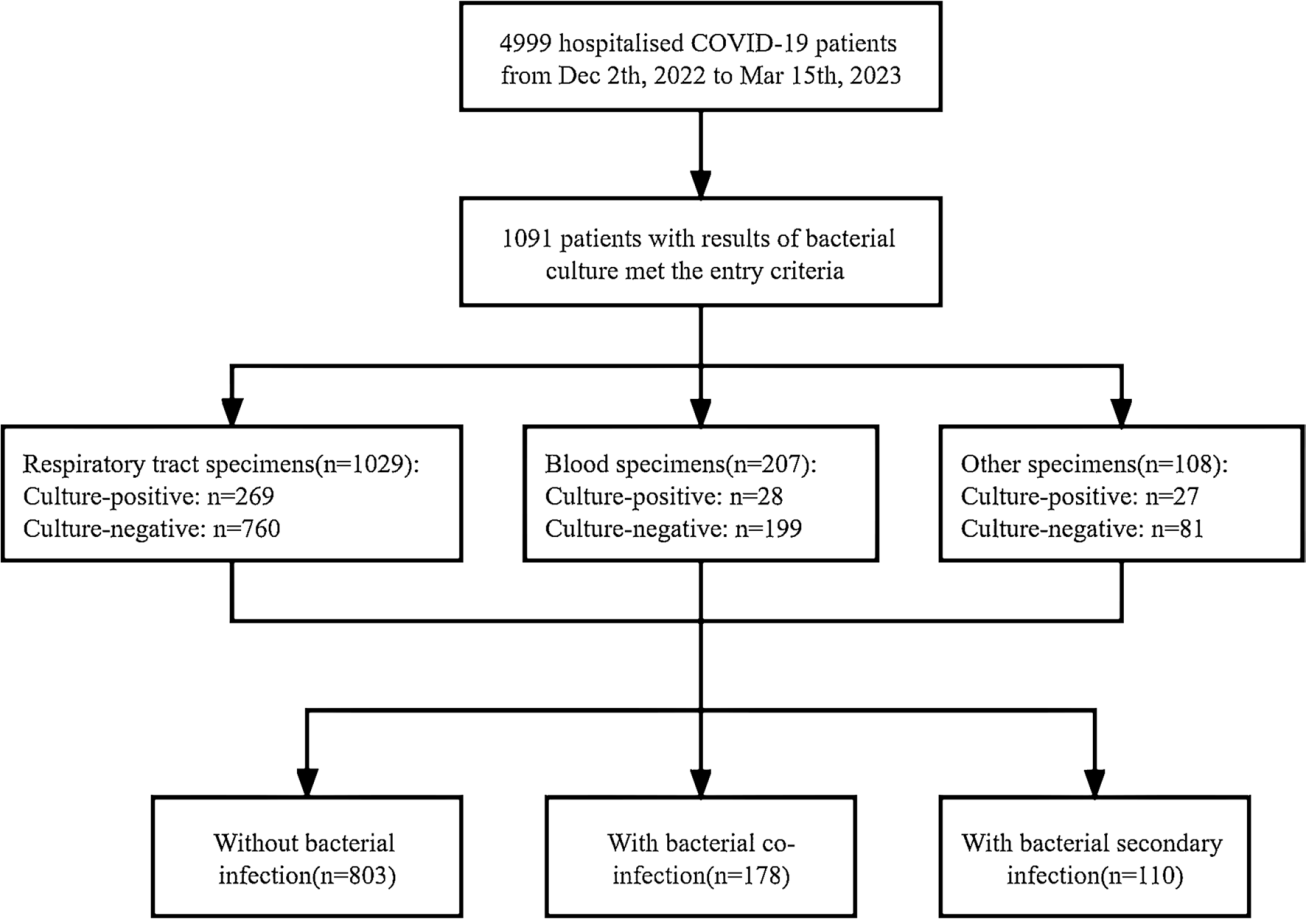
Flowchart of patients’ inclusion

A total of 4999 patients with laboratory-confirmed COVID-19 were admitted to West China Hospital during the period from December 2nd, 2022, to March 15th, 2023. Of these, 1091 patients (including 1029 patients with respiratory tract specimens, 207 patients with blood specimens, and 108 patients with blood specimens) with qualified bacterial culture results were involved in the final analysis (Fig. 1). After further evaluation, the COVID-19 subgroup without bacterial infection consisted of 803 patients. Of these remaining 288 (26.4%) patients complicated with bacterial infection, 178 (16.3%) patients and 110 (10.1%) patients were categorized as bacterial co-infection and secondary infection respectively.

Baseline variates, laboratory parameters, clinical specimens for bacterial culture, and antibiotic prescriptions for patients in three COVID-19 subgroups are listed in Table *Clinical characteristics for COVID-19 patients with bacterial co-infection and secondary infection.*

Baseline information comparison between COVID-19 without bacterial infection and with bacterial co-infection observed no differences in age, gender, BMI, and current or former smoker, but morbidity of chronic lung diseases (27.5% vs 20.5%, *p* = 0.045) and cardiovascular (34.3% vs 23.3%, *p* = 0.002) disease in patients with bacterial co-infection was higher than that of patients without bacterial infection. Advanced age (74.20 ± 14.47 vs 69.53 ± 17.33, *p* = 0.002) and male (80.9% vs 67.5%, *p* = 0.006) were more common in the COVID-19 subgroup with secondary infection than patients without bacterial infection.

Bacterial co-infection and secondary infection rates increased with disease severity of COVID-19. The incidence of bacterial co-infection in non-severe COVID-19, severe COVID-19, and critical COVID-19 subgroups was identified to be 13.0% (56/430), 16.5% (70/424), and 21.9% (52/237). In addition, 4.0% (17/430), 9.0% (38/424), and 23.2% (55/237) patients had bacterial secondary infections in non-severe COVID-19, severe COVID-19, and critical COVID-19 subgroups.

In comparison to patients without bacterial infection, patients with bacterial co-infection had a significantly higher serum PCT, CRP, and lactic dehydrogenase (LDH) level at admission (*p* < 0.05). Similarly, all common infection indicators at admission (including serum WBC count, PCT, CRP, IL-6, D-Dimer, and LDH level) in COVID-19 subgroup with secondary infection were great higher than that of patients without bacterial infection (*p* < 0.05).

As for antibiotic prescription in COVID-19 patients, it is worth noting that as high as 91.5% (735/803) of hospitalized patients with negative bacterial culture results had received antibiotic therapy in our study.

### Impact of bacterial co-infection and secondary infection on clinical outcomes of hospitalized COVID-19 patients

In-hospital mortality of COVID-19 patients with co-infection (17.4% vs 9.5%, *p* = 0.003) and secondary infection (28.2% vs 9.5%, *p* < 0.001) was 17.4% and 28.2% respectively, which greatly exceeded that of COVID-19 patients without bacterial infection (Table 1). Likewise, more patients with co-infection and secondary infection needed mechanical ventilation (37.1% vs 25.5%, *p* < 0.001; 55.5% vs 25.5%, *p* < 0.001), tracheal intubation (15.7% vs 6.6%, *p* < 0.001; 27.3% vs 6.6%, *p* < 0.001), and ICU admission (21.3% vs 8.2%, *p* < 0.001; 43.6% vs 8.2%, *p* < 0.001) than patients without bacterial infection. In comparison with patients without bacterial infection, patients with secondary infection had longer hospital stays (22.06 ± 12.96 vs 15.93 ± 9.34, *p* = 0.002). However, bacterial co-infection did not prolong hospital stays.

**Table 1:** Clinical characteristic and outcomes of patients hospitalized for COVID-19 in three subgroup

	COVID‐19 without bacterial infection (*n* = 803)	COVID‐19 with bacterial co-infection (*n* = 178)	COVID‐19 with bacterial secondary infection (*n* = 110)	p* value	*p*^∆^ value
Baseline covariates					
Age (years)	69.53 ± 17.33	72.26 ± 17.48	74.20 ± 14.47	0.058	0.002
Gender:				0.534	0.006
Female	32.5% (261/803)	29.8% (53/178)	19.1% (21/110)		
Male	67.5% (542/803)	70.2% (125/178)	80.9% (89/110)		
Smoking history	21.8% (175/803)	24.2% (43/178)	28.2% (31/110)	0.550	0.144
Comorbidities:					
Diabetes	25.8% (207/803)	29.8% (53/178)	28.2% (31/110)	0.302	0.643
Hypertension	44.1% (354/803)	43.3% (77/178)	47.3% (52/110)	0.868	0.541
Chronic kidney disease	25.8% (204/803)	28.1% (50/178)	27.3% (30/110)	0.508	0.727
Chronic hepatic disease	6.4% (51/803)	7.9% (14/178)	5.5% (6/110)	0.504	0.836
Chronic lung diseases	20.5% (165/803)	27.5% (49/178)	19.1% (21/110)	0.045	0.801
Cardiovascular disease	23.3% (187/803)	34.3% (61/178)	30.9% (34/110)	0.002	0.096
Immune system disease	4.0% (32/803)	3.4% (6/178)	4.5% (5/110)	0.832	0.795
Malignancy	14.6% (117/803)	11.2% (20/178)	16.4% (18/110)	0.283	0.667
Disease severity for COVID-19:				< 0.001	< 0.001
Non-severe COVID-19	83.0% (357/430)	13.0% (56/430)	4.0% (17/430)		
Severe COVID-19	74.5% (316/424)	16.5% (70/424)	9.0% (38/424)		
Critical COVID-19	54.9% (130/237)	21.9% (52/237)	23.2% (55/237)		
Laboratory parameters at admission					
WBC (10^9^/L)	7.50 ± 4.39	8.22 ± 4.50	9.38 ± 5.30	0.052	0.001
PCT (ng/mL)	0.60 ± 2.12	1.53 ± 3.93	1.84 ± 4.80	0.004	0.012
CRP (mg/L)	66.23 ± 67.06	87.94 ± 75.94	112.76 ± 94.54	< 0.001	< 0.001
IL-6 (μg/L)	51.36 ± 100.96	71.32 ± 126.46	98.02 ± 159.59	0.069	0.005
D-Dimer (mg/L)	3.05 ± 5.17	3.88 ± 6.05	6.41 ± 8.41	0.080	< 0.001
LDH (IU/L)	305.88 ± 136.85	332.54 ± 158.85	410.08 ± 195.83	0.028	< 0.001
Clinical specimens for bacterial culture					
Respiratory tract sample	94.6% (760/803)	97.2% (173/178)	87.3% (96/110)	NA	NA
Blood sample	22.3% (179/803)	4.4% (8/178)	18.2% (20/110)	NA	NA
Other sample	10.1% (81/803)	9.6 (17/178)	9.1% (10/110)	NA	NA
Antibiotic prescription					
Antibiotic within 48 h	89.4% (718/803)	93.3% (166/178)	94.5% (104/110)	NA	NA
Antibiotic after 48 h	2.1% (17/803)	3.4% (6/178)	3.6% (4/110)	NA	NA
Without antibiotic	8.5% (68/803)	3.4% (6/178)	1.8% (2/110)	NA	NA
Outcomes parameters					
Mechanical ventilation	25.5% (205/803)	37.1% (66/178)	55.5% (61/110)	< 0.001	< 0.001
Tracheal intubation	6.6% (53/803)	15.7% (28/178)	27.3% (30/110)	< 0.001	< 0.001
ICU admission	8.2% (66/803)	21.3% (38/178)	43.6% (48/110)	< 0.001	< 0.001
Mortality	9.5% (76/803)	17.4% (31/178)	28.2% (31/110)	0.003	< 0.001
Length of hospital stay (days)	15.93 ± 9.34	16.25 ± 11.32	22.06 ± 12.96	0.694	0.002

p* value, comparison between COVID‐19 patients without bacterial infection and with bacterial co-infection; *p*^*∆*^* value*, comparison between COVID‐19 patients without bacterial infection and with bacterial secondary infection; *BMI*, body mass index; *WBC*, white blood cell; *PCT*, procalcitonin; *CRP*, C-reactive protein; *IL-6*, interleukin 6; *LDH*, lactic dehydrogenase

### Risk factors for bacterial co-infection and secondary infection in hospitalized COVID-19 patients

Variables in the MLR model assessing risk factors for bacterial co-infection included age, chronic lung diseases, cardiovascular disease, disease severity for COVID-19, and serum inflammatory biomarkers (Table 2). Cardiovascular disease (1.847 (1.202–2.837), *p* = 0.005), severe COVID-19 (1.694 (1.033–2.778), *p* = 0.037), and critical COVID-19 (2.220 (1.196–4.121), *p* = 0.012) were proved to be significantly associated with bacterial co-infection. With the same method, our result observed that only critical COVID-19 (1.847 (1.202–2.837), *p* = 0.005) was considered as a risk factor for secondary infection (Table 3).

**Table 2:** The MLR model for risk factors of bacterial co-infection in hospitalized COVID-19 patients

	Exp(*B*)	95.0% CI		*p* value
Age (years)	1.003	0.991	1.015	0.634
Chronic lung diseases	1.202	0.748	1.932	0.447
Cardiovascular disease	1.847	1.202	2.837	0.005
Disease severity for COVID-19*:				0.030
Non-COVID-19 (referent)				
Severe COVID-19	1.694	1.033	2.778	0.037
Critical COVID-19	2.220	1.196	4.121	0.012
WBC (10^9^/L)	0.995	0.949	1.044	0.851
PCT (ng/mL)	1.016	0.934	1.104	0.717
CRP (mg/L)	1.001	0.998	1.005	0.548
IL-6 (μg/L)	1.000	0.999	1.002	0.668
D-Dimer (mg/L)	0.996	0.957	1.037	0.849
LDH	1.000	0.998	1.001	0.805

Disease severity for COVID-19*, take non-severe COVID-19 as a reference; *MLR*, multivariable logistic regression; *WBC*, white blood cell; *PCT*, procalcitonin; *CRP*, C-reactive protein; *IL-6*, interleukin 6; *LDH*, lactic dehydrogenase

**Table 3:** The MLR model for risk factors of bacterial secondary infection in hospitalized COVID-19 patients

	Exp(*B*)	95.0% CI		*p* value
Age (years)	1.012	0.995	1.030	0.159
Gender	0.642	0.351	1.176	0.151
Cardiovascular disease	1.183	0.666	2.103	0.566
Disease severity for COVID-19*:				0.000
Non-COVID-19 (referent)				
Severe COVID-19	1.795	0.819	3.935	0.144
Critical COVID-19	5.465	2.407	12.407	0.000
WBC (10^9^/L)	0.993	0.939	1.051	0.818
PCT (ng/mL)	1.049	0.971	1.133	0.225
CRP (mg/L)	1.002	0.999	1.006	0.221
IL-6 (μg/L)	1.000	0.998	1.002	0.921
D-Dimer (mg/L)	1.021	0.983	1.060	0.290
LDH	1.001	1.000	1.003	0.133

Disease severity for COVID-19*, take non-severe COVID-19 as a reference; *MLR*, multivariable logistic regression; *WBC*, white blood cell; *PCT*, procalcitonin; *CRP*, C-reactive protein; *IL-6*, interleukin 6; *LDH*, lactic dehydrogenase

### Bacterial pathogens detected in respiratory tract samples

**Fig. 2:**
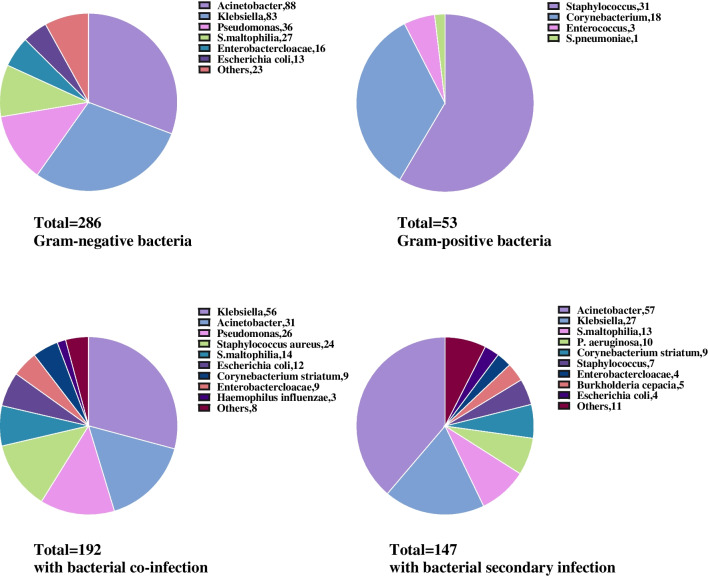
Bacterial isolates detected in respiratory-tract sample

Among 1029 patients with respiratory samples bacterial cultures, a total of 339 bacterial species were found in 26.1% (269/1029) patients. We concluded that Gram-negative (G −) bacterial infection was 84.4% (286/339) and Gram-positive (G +) was 15.6% (53/339). The most common bacteria detected in the respiratory tract include *Acinetobacter*, *Klebsiella*, *Pseudomonas*, *Staphylococcus*, and *Stenotrophomonas maltophilia* (Table 4). Furtherly, *Staphylococcus*, *Corynebacterium striatum*, *Enterococcus faecium*, and *Streptococcus pneumoniae* were frequently detected as G + bacteria that grow in the respiratory tract. *Acinetobacter*, *Klebsiella*, *Pseudomonas*, *Stenotrophomonas maltophilia*, and *Enterobactercloacae* were five of the most common G − bacterias (Fig. 2, detailed information shown in supplement Table S1 and Table S2).

**Table 4:** Microbial etiology in respiratory tract of 269 COVID-19 patients with bacterial infection

Bacterial isolates	*n* (*n*/*N*)
Acinetobacter:	
A. baumanii	73/339 (21.5%)
Other Acinetobacter	15/339 (44.3%)
Klebsiella	
K. pneumoniae	69/339 (20.4%)
Other Klebsiella	14/339 (4.1%)
Pseudomonas	
P. aeruginosa	34/339 (10.0%)
Other Pseudomonas	2/339 (0.6%)
Staphylococcus	
Staphylococcus aureus	29/339 (8.6%)
Other Staphylococcus	2/339 (0.6%)
Stenotrophomonas maltophilia	27/339 (8.0%)
Corynebacterium striatum	18/339 (5.3%)
Enterobactercloacae	16/339 (4.7%)
Escherichia coli	13/339 (3.8%)
Burkholderia cepacia	5/339 (1.5%)
Haemophilus influenzae	4/339 (1.2%)
Serratia marcescens	3/339 (0.9%)
Enterococcus faecium	3/339 (0.9%)
Others	12/339 (3.5%)

A. Baumanii, Acinetobacter baumannii; K. pneumoniae, Klebsiella pneumoniae; P. aeruginosa, Pseudomonas aeruginosa

The following subgroup analysis observed that three common types of respiratory-tract G − bacteria detected within 48 h and after 48 h were exactly the same, including *Acinetobacter*, *Klebsiella*, and *Pseudomonas*. The difference was that the most common G + bacteria in COVID-19 patients with bacterial co-infection were *Staphylococcus aureus*, while *Corynebacterium striatum* was more common in patients with secondary infection (Fig. 2, detailed information shown in supplement Table).

### Bacterial pathogens detected in blood culture

Of those 207 patients who had blood cultures during hospitalization admission, 28 (13.5%) patients with positive blood culture results had found 40 bacterial species. However, nine of the positive results were likely due to skin contaminants, and only 19 (9.2%) were suggestive of clinically significant bacterial infections. These blood cultures were frequently positive for *Staphylococcus*, *Acinetobacter baumannii*, *Klebsiella pneumoniae*, and *Enterococcus faecium* (Table S4).

## Discussion

Above all, the actual bacterial culture-positive rate of various clinical specimens was 16.3% (≤ 48 h) and 10.1% (> 48 h) respectively in our study, which was obviously lower than the antibiotic usage rate among hospitalized COVID-19 patients. Moreover, both bacterial co-infection and secondary infection indicated worse clinical outcomes, including increased risk of death, ICU admission, mechanical ventilation, and tracheal intubation. Further MLR analysis among COVID-19 patients found that cardiovascular disease, severe, and critical COVID-19 were potential risk factors for bacterial co-infection, but only critical COVID-19 was proved to be associated with secondary infections during hospitalization. Lastly, *Acinetobacter*, *Klebsiella*, *Pseudomonas*, *Staphylococcus*, and *Stenotrophomonas maltophilia* were most commonly identified bacterial pathogens in the respiratory tract and blood cultures were frequently positive for *Staphylococcus*, *Acinetobacter baumannii*, *Klebsiella pneumoniae*, and *Enterococcus faecium*.

Here, we reported 16.3% (95%CI 13.0–21.9%) of COVID-19 patients with bacterial co-infection, which was slightly higher than the previously reported bacterial co-infection rate among mixed COVID population with a high degree of heterogeneity [7, 8, 16, 17]. In a meta-analysis, bacterial co-infection and secondary infections were identified in 3.5% and 14.3% of patients respectively [18]. Another retrospective cohort study consisting of 3834 COVID-19 patients found that 7% of patients had a bacterial infection and a significantly increased incidence of bacterial infection (14%) was observed in the ICU ward [8, 16]. The rate of secondary infection was 10.1% in our study, which was consistent with that of previous reported studies [18, 19]. In COVID-19 patients, the overall proportion of bacterial infection was lower than that of the Influenza pandemic, but the antibiotic utilization rate had remained stubbornly high [20, 21]. Nearly 90.0% of patients received empirical antibiotic prescriptions as soon as hospitalization admission in our COVID-19 cohort. However, there was insufficient evidence to support the widespread use of empiric antibiotics in hospitalized COVID-19 patients, especially those non-critically COVID-19 [22].

Our study was conducted after the relaxation of stringent public health measures for COVID-19 in China and viral in this study mainly included the COVID-19-Omicron variant for the study period limited from December 2022 to March 2023. However, 9.5% in-patient hospital mortality in our study higher than generally reported Omicron mortality. The higher mortality and bacterial co-infection rate could be explained by the fact that our analysis excluded asymptomatic COVID-19 patients and the majority of mild COVID-19 patients without urgent need for hospitalization. Consequently, the composition of severe and critical COVID-19 in our patient population was as high as 66.7%, so the mortality and bacterial co-infection rate is more in keeping with studies based on the ICU population. Besides, a rapid surge in the number of cases in a short time imposed huge societal healthcare burdens and resulted in temporary deficiency of medical resources, which were another reason for higher mortality rate in our cohort.

Our study demonstrates an increased risk of worse clinical outcomes (including ICU admission, mechanical ventilation, tracheal intubation, and in-hospital mortality) conferred by both COVID-19 bacterial co-infection and secondary infection, which was inconsistent with a large multicenter retrospective study of 13,781 COVID-19 inpatient encounters [23]. Prompt recognition and early antimicrobial therapy of bacterial infection in hospitalized COVID-19 patients may mitigate the risk of less favorable clinical outcomes [24]. However, excessive and unnecessary antibiotic use could pose a threat to increasing global antibiotic resistance, so there is an urgent need for precise definitions and appropriate predictive biomarkers for the COVID-19 population at high risk of bacterial infection [25]. Common factors associated with bacterial infection have been incorporated in the follow-up analysis to explore possible biomarkers for co-infection and secondary infection in the setting of COVID-19. As one of the reported risk factors for CAP in the Infectious Diseases Society of America (IDSA) Guidelines, chronic cardiovascular disease was also identified to be associated with bacterial co-infection in COVID-19 patients [26]. Besides, severe and critical COVID-19 subtypes also were another risk factor for bacterial co-infection in the MLR analysis, which was consistent with results in another case–control study [27]. As for critical COVID-19, we need to pay extra attention to avoid the occurrence of secondary infections during hospitalization. Both bacterial co-infection and secondary infection rates increased with the severity of COVID-19 in our study, which was consistent with previously reported researches [19, 28]. Overall bacterial infection (including co-infection and secondary infection) in the critical COVID-19 subgroup was as high as 45.1%, so standard empirical antibiotics were recommended for critically ill COVID-19 rather than all the inpatients [25, 29].

The role of serum infection biomarkers in bacterial infection diagnosis among patients hospitalized with COVID-19 remains a controversial topic [10, 11]. Pro-inflammatory cytokines were reported to have applications in risk assessment, monitoring of disease progression and prognosis of COVID-19 patients [12], and our preliminary results showed significantly higher serum inflammatory biomarker levels at admission among COVID-19 with co-infection and secondary infection. However, all common inflammatory biomarkers included in our research have failed to differentiate between pure viral and bacterial infection in subsequent MLR analysis. The SARS-CoV-2 invasion is able to activate the pro-inflammatory response and cause the subsequent release of a large number of inflammatory factors, so it is not reliable enough to reveal whether the infection is viral or bacterial by serum inflammatory biomarkers [10, 30].

Nasopharyngeal bacteria, such as *Streptococcus pyogenes* and *Streptococcus pneumoniae*, have been reported as the most common bacterial pathogens detected in influenza patients due to the physical barrier of the respiratory tract damaged by influenza attacks [2]. Similarly, *Staphylococcus* also was the most common G + bacteria in COVID-19-associated respiratory tract infections and bloodstream infections. G − bacteria (84.4%) are the most frequently detected bacterial isolates among COVID-19 patients, which was consistent with previously reported data [7, 31]. Different from the pathogens in CAP and influenza, we found G − bacteria frequently identified both within 48 h and after 48 h in the setting of COVID-19 were *Acinetobacter*, followed by *Klebsiella*, *Pseudomonas*, which was similar to reported bacteria implicated in hospital-acquired infections [8, 31, 32]. Our findings on common bacteria-caused co-infection and secondary infections are essential to promote antibiotic reasonable application.

Although our research assessed the association between bacterial infection and COVID-19, it has several limitations worth noting. Firstly, this was a single-center study, and our result based on hospitalized adult patients (a high proportion of severe and critical COVID-19) may not reflect the overall bacterial infection rates as the vast majority of COVID-19 patients experience mild disease and do not require hospitalization. Secondly, diagnosis of bacterial infection depended on bacterial culture and ignored other etiological detection methods. Moreover, it is difficult to distinguish bacterial colonization from infection in the setting of COVID-19 infection. Lastly, as some patients received antibiotics before admission, this was unavoidable that some secondary infections were wrongly classified as co-infections.

## Conclusion

Above all, antibiotic utilization rate in clinical work far outstripped the actual incidence of bacterial co-infection and secondary infection in COVID-19 patients, but its presence could aggravate the disease severity and worsen clinical outcomes of COVID-19 patients. In MLR analysis, only critical COVID-19 subtype was identified to be an independent risk factor for both co-infection and secondary infection. So our result supported standard empirical antibiotics for critically ill COVID-19 rather than all the inpatients. Moreover, *Acinetobacter*, *Klebsiella*, and *Pseudomonas* were the most common pathogens to cause respiratory tract infections in COVID-19 patients.

### Supplementary Information

Below is the link to the electronic supplementary material.

Supplementary file1 (DOCX 20 KB)

## Data Availability

The data presented in this study are available on request from the corresponding author.
