# Supplementary material for: Risk factors, outcomes, and epidemiological and etiological study of hospitalized COVID-19 patients with bacterial co-infection and secondary infections
Source: Eur J Clin Microbiol Infect Dis. 2024 Jan 22;43(3):577–86. doi: 10.1007/s10096-024-04755-5 (PMC10917871; doi:10.1007/s10096-024-04755-5)
Supplement: Supplementary file 1 — Supplementary file1 (DOCX 20 KB) [file 10096_2024_4755_MOESM1_ESM.docx]

**TableS1. Gram-negative bacteria detected in respiratory-tract samples.**

| Bacterial isolates | n（n/N） |
| --- | --- |
| Acinetobacter： | 88/286(30.8%) |
| *A. baumanii* | 73/286(25.5%) |
| Other Acinetobacter | 15/286(5.3%) |
| Klebsiella | 83/286(29.0%) |
| *K. pneumoniae* | 69/286(24.1%) |
| Other Klebsiella | 14/286(4.9%) |
| Pseudomonas | 36/286(12.6%) |
| *P. aeruginosa* | 34/286(11.9%) |
| Other Pseudomonas | 2/286(0.7%) |
| *Stenotrophomonas maltophilia* | 27/286(9.4%) |
| *Enterobactercloacae* | 16/286(5.6%) |
| *Escherichia coli* | 13/286(4.5%) |
| *Burkholderia cepacia* | 5/286(1.7%) |
| *Haemophilus influenzae* | 4/286(1.4%) |
| *Serratia marcescens* | 3/286(1.0%) |
| Others | 11(3.8%) |

*A. Baumanii: Acinetobacter baumannii; K. pneumoniae: Klebsiella pneumoniae;  P. aeruginosa: Pseudomonas aeruginosa.*

**TableS2. Gram-positive bacteria detected in respiratory-tract samples.**

| Bacterial isolates | n（n/N） |
| --- | --- |
| Staphylococcus | 31/53(58.5%) |
| *Staphylococcus aureus* | 29/53(54.7%) |
| Other Staphylococcus | 2/53(3.8%) |
| *Corynebacterium striatum* | 18/53(34.0%) |
| *Enterococcus faecium* | 3/53(5.7%) |
| *Streptococcus pneumoniae* | 1/53(1.9%) |

**TableS3. Bacterial co-infection and secondary infection pathogens in respiratory-tract sample.**

| Bacterial isolates | bacterial infection n=192 | secondary infection n=147 |
| --- | --- | --- |
| Acinetobacter | 31(16.1%) | 57(38.8%) |
| *A. baumanii* | 25(13.0%) | 48(32.7%) |
| Other Acinetobacter | 6(3.1%) | 9(6.1%) |
| Klebsiella | 56(29.2%) | 27(18.4%) |
| *K. pneumoniae* | 48(25.0%) | 21(14.3%) |
| Other Klebsiella | 8(4.2%) | 6(4.1%) |
| Pseudomonas | 26(13.5%) | 10(6.8%) |
| *P. aeruginosa* | 24(12.5%) | 10(6.8%) |
| Other Pseudomonas | 2(1.0%) | 0(0.0%) |
| Staphylococcus | 24(12.5%) | 7(4.8%) |
| *Staphylococcus aureus* | 24(12.5%) | 5(3.4%) |
| Other Staphylococcus | 0(0.0%) | 2(1.4%) |
| *Stenotrophomonas maltophilia* | 14(7.3%) | 13(8.8%) |
| *Escherichia coli* | 12(6.3%) | 4(2.7%) |
| *Enterobactercloacae* | 9(4.7%) | 4(2.7%) |
| *Corynebacterium striatum* | 9(4.7%) | 9(6.1%) |
| *Haemophilus influenzae* | 3(1.6%) | 1(0.7%) |
| *Enterococcus faecium* | 2(1.0%) | 1(0.7%) |
| *Others* | 7(3.6%) | 10(6.8%) |

*A. Baumanii: Acinetobacter baumannii; K. pneumoniae: Klebsiella pneumoniae;  P. aeruginosa: Pseudomonas aeruginosa.*

**TableS4. Microbial etiology in blood of COVID-19 patients with bacterial infection.**

| Bacterial isolates | n（n/N） |
| --- | --- |
| *Staphylococcus* | 9/40(22.5%) |
| *A. baumanii* | 11/40(27.5%) |
| *K. pneumoniae* | 5/40(12.5%) |
| *Enterococcus faecium* | 5/40(12.5%) |
| *Corynebacterium* | 2/40(5.0%) |
| *Stenotrophomonas maltophilia* | 2/40(5.0%) |
| *Enterobactercloacae* | 2/40(5.0%) |
| *Serratia marcescens* | 1/40(2.5%) |
| *Escherichia coli* | 1/40(2.5%) |
| *Burkholderia cepacia* | 1/40(2.5%) |
| *faecalis bacteria* | 1/40(2.5%) |

*A. Baumanii: Acinetobacter baumannii; K. pneumoniae: Klebsiella pneumoniae.*
